# Are Baby Shows Vulgar and Valueless?

**Published:** 1917-07-21

**Authors:** 


### ARE BABY SHOWS VULGAR AND VALUELESS?

SOCIAL agitations which aim at popularity are very apt to conclude that every means is good which secures a crowd. The end is seen with such generous eyes that everything which seems to contribute to it appears to justify a welcome. Excellent intentions are no guarantee against this seduction, and even really good people have been known to yield to it. We are afraid that recent attempts to excite the interest of the nation in the preservation of infant life have not altogether escaped this danger. No one questions the desirability of the object, nor the motives of those who minister to it. But we think that discretion, sound judgment, and good taste cannot be detected in that part of the agitation which is concerned with the organisation of "Baby Shows." In these, it would appear, mothers are invited to conduct their offspring to some public hall and to submit the infants to a scheme of classification based upon standards which, so far as we know, remain without definition. The precedent of the cattle show is closely followed. Public announcements herald the event, and the local celebrities contribute their patronage. Judges are appointed, and proceed to value the "exhibits" in an atmosphere throbbing with the expectations of the exhibitors. Whether maternal testimony to character is admitted we do not know. Finally come the fixing of the labels and the attachment of the ribbons. Then the show is over, and the neighbouring streets witness the decorated infants led home by proud mothers, just as after kindred events they witness champion stallions and prize puppies blazing with trophies. The words of the maternal proprietors of the undecorated infants may be left to the imagination.

What special part in the promotion of infant welfare "shows" of this order are supposed to play we cannot guess. The most conspicuous feature of the performance, to our view, is its essential vulgarity, and next we should place its capacity for the encouragement and promotion of vulgarity. Maternal self-respect, reserve, and dignity and delicacy of mind, are, after all, not unimportant qualities in the rearing and training of children. "Baby Shows" will hardly contribute to their growth. Our contention, indeed, is that such exhibitions will move to quite opposite ends. It is because of this, and because of the public estimation of infants, like so many competitive cattle, that we say these methods must exercise a vulgar and debasing tendency.

There is another feature of the performance which justifies criticism. We refer to the large element of pretence and unreality which must in the nature of things characterise the awards of the "judges." By what precise standards can anyone pretend to appraise the comparative values of the individuals in a group of healthy babies? The pretence to do so is little better than a sham, and is utterly without scientific or practical value. When the actual conditions of the exhibition are tested its real values become still more apparent. Thus we read of one instance in which 659 babies were judged by "three lady doctors" and a hospital matron, eighty mothers with their infants being "admitted at a time" to the judgment chamber. Can a more farcical entertainment be imagined? Sir Alfred Keogh has lately told us that no practitioner can possibly examine more than forty recruits per day. Yet here are four ladies who undertake each to examine and place in accurate groups 160 babies, not to speak of the consultations which for purposes of a joint deliverance they must necessarily have cultivated; and all this amidst the cries of a crowd of infants, and doubtless also much maternal perspiration. That medical practitioners should lend themselves to such an enterprise is not a little surprising. Yet here is the "baby show" in being, and we fancy our readers will agree that it is as valueless as it is vulgar.

